# Perivascular mechanical environment: A narrative review of the role of externally applied mechanical force in the pathogenesis of atherosclerosis

**DOI:** 10.3389/fcvm.2022.944356

**Published:** 2022-10-19

**Authors:** Takashi Yamaguchi, Katsutaro Morino

**Affiliations:** ^1^Yamaguchi Clinic, Otsu, Japan; ^2^Institutional Research Office, Shiga University of Medical Science, Otsu, Japan

**Keywords:** atherosclerosis, site specificity, mechanical force, perivascular environment, adventitia

## Abstract

Atherosclerosis is promoted by systemic factors, such as dyslipidemia, hypertension, diabetes, and smoking, which cause atherosclerosis in blood vessels throughout the body. However, atherosclerotic lesions are characterized by their frequent occurrence in specific vessels and sites. Blood vessels are exposed to various mechanical forces related to blood pressure and flow. Although shear stress promotes the initiation and progression of atherosclerotic lesions, the pathogenesis of site specificity of atherosclerosis is not sufficiently explained by shear stress. We propose the concept of a perivascular mechanical environment (PVME). Compelling evidence suggests that site specificity in atherosclerotic lesions depends on a distinct local PVME. Atheroprone arteries, such as the coronary artery, are markedly affected by externally applied mechanical force (EMF), whereas atheroprotective arteries, such as the internal thoracic artery, are less affected. Recent studies have shown that the coronary artery is affected by cardiac muscle contraction, the carotid artery by the hyoid bone and the thyroid cartilage, and the abdominal aorta and lower extremity arteries by musculoskeletal motion. We speculate that the thoracic cage protects the internal thoracic artery from EMF owing to a favorable PVME. Furthermore, evidence suggests that plaque eccentricity is provided by EMF; plaques are frequently observed on an external force-applied side. In each vascular tree, site-specific characteristics of the PVME differ substantially, inducing individual atherogenicity. From the perspective of the mechanical environment, hemodynamic stress occurs in an inside-out manner, whereas EMF occurs in an outside-in manner. These inward and outward forces apply mechanical load individually, but interact synergistically. The concept of a PVME is a novel pathogenesis of atherosclerosis and also might be a pathogenesis of other arterial diseases.

## Introduction

**FIGURE 1:**
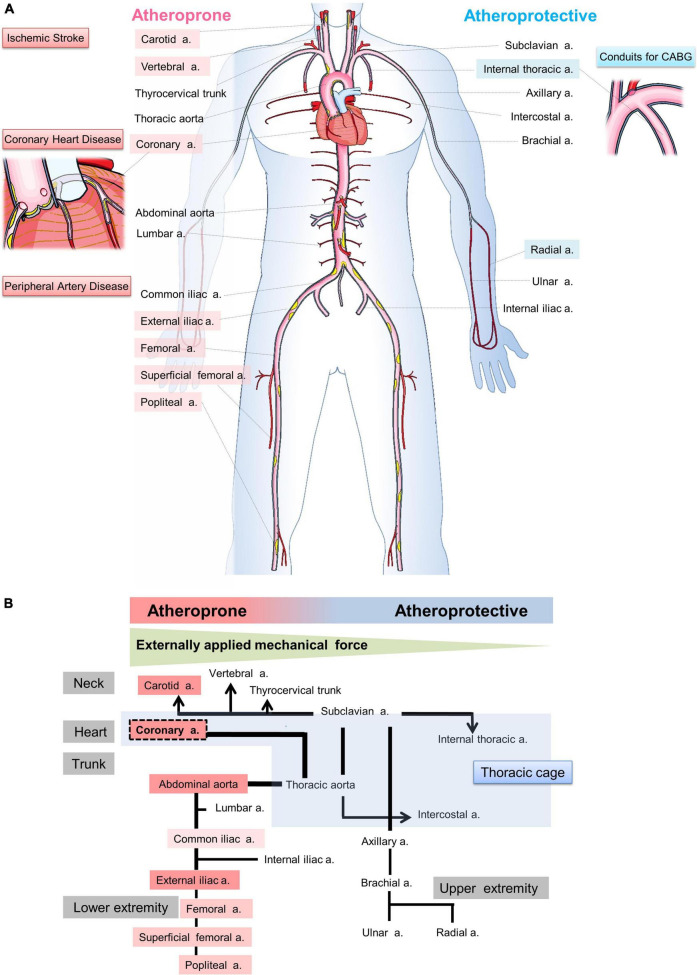
Illustration of hypothesis: Site-specific relationship between the perivascular mechanical environment and atherogenicity. **(A)** Coronary heart disease, ischemic stroke, and peripheral artery disease are major elements of atherosclerosis, and the coronary arteries, carotid arteries, and iliac arteries are popular sites of atherosclerosis. On the other hand, internal thoracic arteries are used for coronary artery bypass grafting because they are less prone to atherosclerosis. The susceptibility to atherosclerosis varies from artery to artery. However, how such differences arise is unclear. **(B)** This figure illustrates the hypothesis that the effect of externally applied mechanical force (EMF) on blood vessels may be important in the development of atherosclerosis due to the different mechanical environments surrounding blood vessels in individual arteries.

Atherosclerosis is a multifocal inflammatory process that mainly affects the heart (coronary heart disease), brain (ischemic stroke), or lower extremities (peripheral artery disease), and its underlying pathogenesis has been reported in excellent reviews in terms of lipoproteins (1), inflammation (2), endothelial dysfunction (3), and hemodynamic shear stress (4). Traditional risk factors, such as dyslipidemia, hypertension, diabetes, and smoking, show systemic effects on all vessels (5). However, the distribution of atherosclerotic lesions varies among each arterial tree (Figure 1A). The coronary, carotid, and lower extremity arteries, and the abdominal aorta are susceptible for atherosclerosis (atheroprone), whereas the internal thoracic artery (ITA) and upper extremity arteries are less prone for atherosclerosis (atheroprotective) (6–9). The pathophysiology of the site specificity is mainly explained by hemodynamic stress including the shear stress (9, 10) but it remains unclarified. Therefore, this review focuses on a different point of view: the possible influence of the perivascular mechanical environment (PVME) on the site specificity of atherosclerosis.

Arterial walls are exposed to mechanical forces such as blood pressure and blood flow. Vascular running and bifurcation cause different hemodynamic stresses within the same artery, and the vascular endothelium is particularly affected by shear stresses. On the other hand, vessels are subjected to external forces depending on the environment in which they are placed. For example, coronary arteries receive extravascular compressive forces from myocardium. Peripheral arteries are mostly located between musculoskeletal tissues (muscle, bone, cartilage and adjacent connective tissue) and receive mechanical forces from these tissues throughout their lifetime. In recent years, compelling evidence has accumulated to support the concept that externally applied mechanical force (EMF) causes atherosclerosis in various site-specific atheroprone situations. However, the concept is not generally recognized and has not comprehensively described yet. Furthermore, there is no description concerning the possibility that atheroprotective arteries are less susceptible to EMF.

We would like to propose that the PVME may play a role in the site-specific pathogenesis of atherosclerosis (Figure 1). In the case of coronary artery, the heart beats approximately 100,000 times per day. The heartbeat, an example of EMF, moves and stretches the coronary artery. We suspect that decades of repeated EMF, in addition to shear stress and classical atherosclerotic risk factors, may promote atherosclerosis as an additional effect.

In this article, we review the concept of PVME-induced atherosclerosis by focusing on site specificity. To examine the possible role of EMF on the progression of atherosclerosis, we review the features of atherosclerosis in each arterial tree.

## Site-specific ultrasound imaging in each arterial tree

**FIGURE 2:**
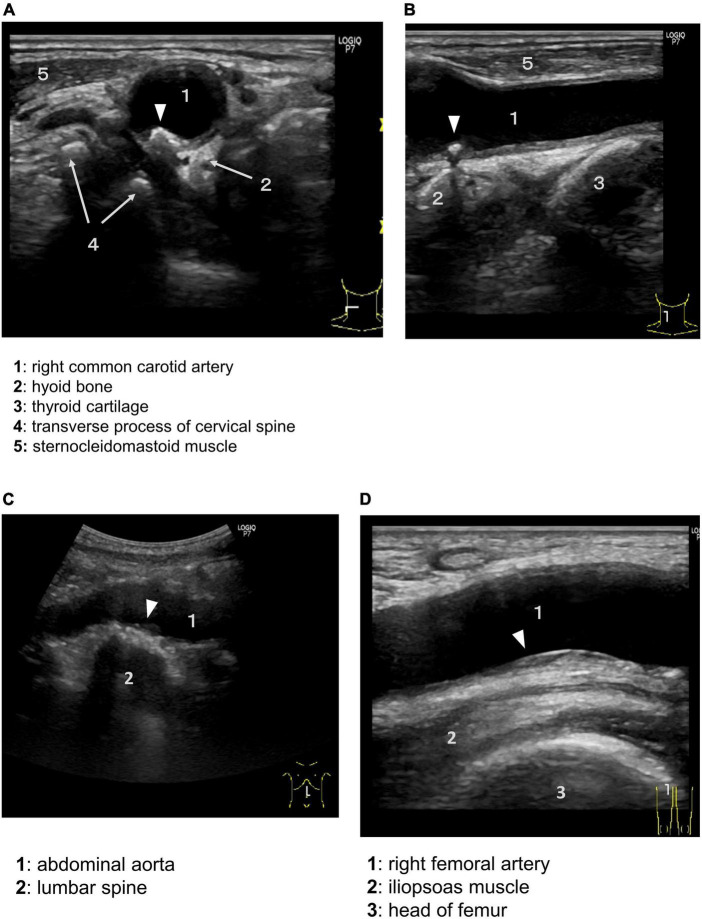
Perivascular environment of the arteries and aorta as shown by ultrasound. Plaques are not uniformly distributed in the vessel wall, but tend to occur in specific areas. Arrowheads indicate atherosclerotic plaque. Axial **(A)** and sagittal **(B)** ultrasound showing the common carotid artery (1) near the bifurcation bounded by the hyoid bone (2), thyroid cartilage (3), transverse process of the cervical spine with anterior and posterior tubercles (4), and sternocleidomastoid muscle (5). **(C)** Abdominal aorta (1) bounded posteriorly by the lumbar spine (2). **(D)** Right femoral artery (1), iliopsoas muscle (2), and head of the femur (3).

Each artery is surrounded by site-specific tissues and organs. As an example of this situation, ultrasounds in the carotid artery (CA), abdominal aorta, and femoral artery of a 79-year-old man are shown (Figure 2). The ultrasound images were taken in a neutral supine position. The common CA near the bifurcation was surrounded by the hyoid bone, thyroid cartilage, transverse process of the cervical spine, and sternocleidomastoid muscle (Figures 2A,B). A calcified plaque was observed near the greater horn of the hyoid bone. The abdominal aorta was located anterior to the vertebral bodies of the lumbar spine (Figure 2C). The femoral artery was bounded posteriorly by the iliopsoas muscle, which is the most powerful muscle of the hip flexors (Figure 2D). Plaques were located in the posterior (far) wall. Neighboring tissues and organs move actively and change their positions relative to the artery during motions in daily life. These observations suggest that EMF induces atherosclerosis. Below, we examine the concept of PVME in each arterial tree.

## Site-specific PVME in each arterial tree

### Heart

The heart pumps blood through vessels to all parts of the body including the heart itself. Contraction and relaxation of the myocardium causes the coronary arteries themselves to move and stretch on the epicardium, the outer surface of the heart. In addition, intramuscular blood vessels are compressed by the contracting myocardium. Eccentric plaques are vulnerable (11). These plaques are more frequently distributed toward the myocardial side than toward the lateral and epicardial sides of the coronary artery, evaluated with intravascular ultrasound (IVUS) (12, 13). EMF by the myocardial contraction may affect the myocardial (inner) side to a greater degree than the epicardial (outer) side. In a previous study, analyses of coronary artery motion, analyzed by coronary angiograms, showed arterial compression and deformity related to atherosclerotic sites (14). The left ventricular wall consists of overlapped subepicardial and subendocardial myocardial layers, which are oriented in a counter-directional helical myocardial structure (15). Left ventricular torsion, which is important for cardiac efficiency, generates opposite rotations of the apex and the base, analyzed by magnetic resonance imaging (15). An epidemiological study, which includes 1,478 participants with multi ethnics, reported that this torsion is greater as age increases and with hypertension (16). We speculate that torsion may lead to atherosclerosis of the coronary artery tethered by the epicardial surface.

A myocardial bridge is a congenital coronary anomaly in which a segment of the epicardial coronary artery traverses through myocardium for portion of its length (17). Recently, a myocardial bridge is thought to be related to not only the focal atherosclerosis but also various cardiovascular events: coronary spasm, coronary dissection, arrhythmia, and sudden cardiac death (17, 18). Almost all myocardial bridges are located in the left ascending artery. A histopathological study with 150 autopsy subjects with myocardial infarction has shown that a myocardial bridge is a consistent cause of myocardial infarction because of its anatomical properties, such as the location, length, and thickness (19). The proximal segment of the myocardial bridge is atheroprone; the pathogenesis is explained by the low wall shear stress. Retrograde flow at the entrance of the myocardial bridge due to systolic compression is considered to create low wall shear stress (17). Conversely, the intramural segment is atheroprotective. A lower susceptibility to atherosclerosis in the intramural segment is explained by limited progress of the adventitial vasa vasorum (20) and the absence of epicardial adipose tissue (21). A reduced coronary wall motion due to external support of the surrounding myocardium may also play a role in the pathogenesis of atherosclerosis (22).

### Neck

The neck joins the head to the trunk and extremities and serves as a major conduit for structures passing between them. The inflow arteries, such as the CA and the vertebral artery, are affected by mechanical force arising from neck rotation and bending. The vertebral artery at its origin from the superior aspect of the subclavian artery is atheroprone (23), whereas the portion of this artery passing through the bone canal is free from atherosclerotic lesions (24). Evidence suggests that PVME is related to the pathogenesis of carotid atherosclerosis. The common CA ascends anterolaterally to the cervical spine behind the sternocleidomastoid muscle to the upper edge of the thyroid cartilage of the larynx, where it divides to form the internal and external CA (25). A study using magnetic resonance imaging, the geometry of carotid bifurcation varies depending on aging and the atherosclerotic process (26). The hyoid bone and the thyroid cartilage move together during swallowing (27). The displacement of the carotid bifurcation is observed during swallowing (28). Moreover, the hyoid bone moves during speech in conjunction with the tongue (29).

Recent studies have highlighted that the position of the hyoid bone is an important mechanism of atherogenicity of the internal CA. A study using computed tomography angiograms showed that the proximity of the hyoid bone to the internal CA is a risk factor for CA dissection (30). The displacement of the internal CA to and from a retropharyngeal position has been reported in several studies (31–34). Kinoshita et al. reported that displacement of the internal CA was related to the internal CA stenosis (32). Moreover, a case report showed that mechanical stimulation by the thyroid cartilage is proposed to induce a plaque during frequent neck rotation (35). Another case report showed that embolic retinal and brain ischemia were caused by mechanical compression of the internal CA by the greater horn of the hyoid bone and the superior horn of the thyroid cartilage (29). These cases were diagnosed by dynamic three-dimensional computed tomography angiography during swallowing. We also occasionally encounter similar imaging characteristics by routine ultrasound (Figures 2A,B). Consequently, the carotid bifurcation is compressed and rubbed by the neighboring cervical spine, sternocleidomastoid muscle, hyoid bone, and thyroid cartilage during head rotation, swallowing, and speech. This region is in a much more hostile PVME than other arteries, such as the intracranial arteries. These mechanical contacts are suspected to induce atherosclerosis.

### Trunk

In the trunk, the infrarenal abdominal aorta is atheroprone, while the ITA is atheroprotective. The segmental arteries, namely the intercostal arteries and lumbar arteries, have site-specific differences in atherogenicity. The lumbar arteries are much more atheroprone than the intercostal arteries. The proximal aorta is susceptible to mechanical force from the motion of the contracting heart. This mechanical force provokes ascending aortic dissection and aneurysms. However, genetic influences play a more prominent role in thoracic aortic aneurysms (36), and abdominal aortic aneurysms are primarily caused by atherosclerosis. In the aorta, atherosclerotic lesions are more affected in the abdominal aorta, especially in the infrarenal aorta, than in the thoracic aorta. The mechanism underlying each site-specific atherogenicity remains unclear.

Degenerative intervertebral instability is related to the pathogenesis of spinal degenerative disease, hernias, and spinal canal stenosis. This instability occurs in the cervical or lumbar spine and seldom occurs in the thoracic spine (37). The stability of the thoracic spine is related to a lower ability of flexion owing to the presence of the thoracic cage (rib cage), which is composed of the spine, ribs, and sternum (37, 38). Except for the ascending aorta, which is susceptible to the beating heart, intrathoracic arteries, such as the descending aorta, the ITA, and intercostal arteries, are less influenced by EMF than other arteries owing to the support of the thoracic cage (Figure 1).

The infrarenal aorta is subjected to mechanical force of the musculoskeletal system that is induced during hip flexion (39). The adventitia of the abdominal aorta is suspected to be affected by mechanical force from the adjacent vertebral body and psoas muscle. A rupture of an abdominal aortic aneurysm tends to be located on the posterior or posterolateral side (40), which is adjacent to the vertebral body and iliopsoas muscle. Osteophytes that frequently form in the lumbar spine are related to the atherogenesis of an abdominal aortic aneurysm (41, 42). Aortic atherosclerosis and stenosis of the feeding arteries in the lumbar spine reduce blood flow, resulting in disk degeneration and lower back pain (43). In an abdominal aortic aneurysm, the presence of an intraluminal thrombus frequently occludes the orifices of the lumbar arteries (44). Taken together, these findings suggest that the abdominal aorta, especially the infrarenal aorta, is vulnerable to the mechanical force of skeletal motion. Therefore, this atheroprone region is in a hostile PVME.

The ITA is selected for the first-line conduit in coronary artery bypass grafting. The remarkable atheroprotective properties of the ITA are explained by its beneficial endothelial properties, such as a higher nitric oxide production (45), and the beneficial phenotype of perivascular adipose tissue (PVAT) surrounding the ITA for chronic inflammation and adipose tissue remodeling (46). Numaguchi et al. discussed the mechanical properties of the ITA originating from the subclavian artery where it exists in a stable retrosternal position (46). The ITA originates from the lower aspect of the subclavian artery. However, more atheroprone arteries, such as the vertebral artery and the thyrocervical trunk, originate from the upper aspect. Kauppila and Penttilla stated that the origin on the upper aspect of the subclavian artery probably causes the arteries to be vulnerable to arterial disease, but the cause and effect remain unclear (23). We speculate that this site-specific atherogenicity is related to the PVME. The vertebral artery and the thyrocervical trunk, which travel upward through the neck, are subjected to dynamic force induced during neck motion. However, the ITA is protected by the thoracic cage from mechanical force. This atheroprotective artery is located in a favorable PVME. Moreover, in the thoracic cavity, cardiac and respiratory tissue activity promotes lymphatic transport to the neighboring thoracic duct, which is beneficial for reverse cholesterol transport (47). The ITA has considerable advantages of atheroprotection over other arteries.

### Lower extremities

The lower extremities are extensions from the trunk specialized for the support of weight, adaptation to gravity, and locomotion (48). The lower extremity arteries start from the common iliac artery originating from the abdominal aorta and run almost through a long intermuscular passage. There are several atheroprone arteries, which comprise the external iliac artery, femoral artery, superficial femoral artery, and popliteal artery (8, 49, 50). The inflow arteries of the lower extremities (common and external iliac arteries) follow the iliopsoas muscle, which is the most powerful of the hip flexors with the longest range (48). The femoral artery is exposed to musculoskeletal motion of the posteriorly located psoas muscle and usually bends during sitting and walking. The femoral artery is commonly investigated by ultrasound. Atherosclerotic changes are rarer in the near wall of this artery compared with the far wall (51) (Figure 2D). This is inconsistent with hemodynamic theory because hemodynamic stress along the inner curvature (near wall) is higher than that along the outer curvature (far wall). The superficial femoral artery runs through an approximately 15-cm intermuscular passage (adductor canal) (48). This artery undergoes considerable deformation and buckling during hip and knee flexion, resulting in atherosclerosis, stent-vessel interaction, and stent fracture (50). The popliteal artery is affected by unique mechanical compression-related disorders, such as popliteal artery aneurysm, popliteal artery entrapment syndrome, and cystic adventitial disease (52). Popliteal arterial occlusion is induced in approximately half of subjects with simple leg positioning caused by myofascial compression (53). Critical limb ischemia, which is the most serious complication of peripheral artery disease, frequently results from thrombotic occlusion of peripheral vessels (54). An upstream atheroprone aorta and lower extremity arteries are thought to result in downstream atherothrombotic lesions, as observed in cholesterol embolization syndrome.

### Upper extremities

In contrast to the lower extremities and the other areas, overt atherosclerosis of the upper extremity arteries is relatively rare (55). The pathogenesis of this atheroprotective tendency remains to be clarified. The following reasons could explain the lower rate of atherosclerosis of the upper extremity arteries. First, the degree of atherosclerosis in the inflow vessels is different. The inflow arteries of the lower extremities (common and external iliac arteries), which are affected by mechanical load from the lower extremity muscles, are atheroprone. In contrast, the inflow arteries of the upper extremities (subclavian and axillary arteries), which are located in the upper margin of the thoracic cage, are not atheroprone. Second, the muscle volume in the upper extremities is approximately one fifth of that in the lower extremities (56). In contrast to the lower extremities, the upper extremities are not usually involved in weight bearing or motility (48), and therefore, are less affected by mechanical load. Third, the main arteries of the upper extremities, which are the brachial and radial arteries, are relatively superficial and palpable throughout these courses (48). Therefore, these arteries are less affected by compression or irritation of the surrounding muscles than arteries in the lower extremities.

## Role of the PVME in the progression of atherosclerosis: Perspectives for future research directions

**FIGURE 3:**
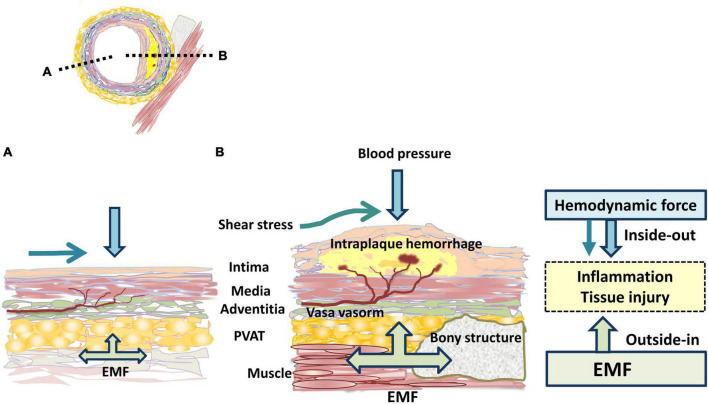
Mechanical force related to atherogenicity. The black dotted lines indicate the location of longitudinal sections shown in the panels **(A,B)**. The atheroprone side **(B)** is proposed to be much more affected by EMF than the atheroprotective side **(A)**. EMF: externally applied mechanical force; PVAT: perivascular adipose tissue.

Atherosclerosis is a chronic inflammatory disease (57) that develops in a specific location in vessels (6–8). Blood vessels are exposed to various mechanical forces related to blood pressure and flow (24, 58, 59). Atherosclerosis occurs mostly in bifurcation, branch points and regions of high curvature that result in disturbed pattern of blood flow. Shear stress promotes the initiation and progression of atherosclerotic lesions and induces plaque destabilization (58). However, the pathogenesis of site specificity of atherosclerosis is not sufficiently explained by shear stress (9, 10, 24). We propose the concept of a site-specific PVME in atherosclerosis (Figure 1B). Atheroprone arteries are markedly affected by EMF, whereas atheroprotective arteries are less affected. Previous studies have reported that the coronary artery is affected by cardiac muscle contraction (13, 14, 18–22), the CA by the hyoid bone and the thyroid cartilage (30–35), and the abdominal aorta (39, 41, 42) and lower extremity arteries (50, 52, 53) by musculoskeletal motion. We speculate that the thoracic cage (37, 38) protects the ITA from EMF because of a favorable PVME. Furthermore, plaques are frequently observed on an external force-applied side, suggesting that EMF provides plaque eccentricity (12, 13). Moreover, the direction that vessels travel and the geometry are regulated by the surrounding tissues and dynamically affected by EMF (31–35). These findings suggest that the PVME is also a determining factor of shear stress. Interindividual variations in the geometry considerably increase with aging and the atherosclerotic process (26). Accordingly, we propose that the PVME is a novel concept that explains the site specificity of atherosclerosis. EMF induces a repetitive compressive load through the adventitia to the inner wall. From the luminal side, hemodynamic force induces stress to the wall. Collectively, externally applied centripetal force and hemodynamic-related centrifugal force exert a dynamic compressive load on the vessel wall to opposite sides (Figure 3). These inward and outward forces apply a mechanical load individually but interact synergistically. Moreover, EMF stretches the vascular wall, inducing tissue injury. The absence of atherosclerosis in the intramyocardial coronary arteries (20–22) and in the intraosseal portions of the vertebral arteries (24) is highly suggestive of the following concept: the vascular wall is more vulnerable to dynamic EMF (e.g., stretch) than to static EMF (e.g., compression). Therefore, in each vascular tree, site-specific characteristics of PVME induce individual atherogenic potential.

Mouse models, especially apoE knockout and LDL receptor knockout mice, are frequently used in atherosclerosis research. The atheroprone sites are the aortic root, the lesser curvature of the aortic arch, and the principal branches of the aorta (60, 61): These vessels are elastic arteries closest to the heart. On the other hand, the atheroprone sites of human atherosclerosis are the abdominal aorta and coronary, carotid, external iliac, femoropopliteal arteries; among them, coronary, external iliac, femoropopliteal artery are muscular arteries. Mouse models suggest that the atherosclerosis caused by severe dyslipidemia in their short life are related to the elasticity of the arterial wall and its embryological origin (62). Indeed, human familial hyperlipidemia homozygotes develop lesions at the aortic root as in the mouse model within the first few years of life. Subsequently, in their 20’s, they develop systemic atherosclerosis, mainly coronary artery lesions (63). These facts suggest that EMF repeated over decades affects atherosclerosis as well as shear stress. Atherosclerosis is an age-related condition; PVME may be an important factor affecting vessels throughout life.

In atherosclerotic lesions, the infiltration of inflammatory cells is considered to enter the vessel wall from the luminal side (inside-out theory) (64). The perivascular environment such as PVAT is being vigorously studied for its impact on atherosclerosis. Recently, vascular inflammation was proposed to be initiated in the adventitia and progress inward (outside-in theory) (65, 66). The adventitia, which is a complex and dynamic compartment of the vessel wall, consists of an extracellular matrix scaffold containing fibroblasts, blood and lymphatic vessels, nerves, and progenitor and immune cells (66). PVAT is located in the outside of the adventitia, lacks a separating fascia (67, 68) and accesses the arterial wall by diffusion or *via* the vasa vasorum (69). From the perspective of the mechanical environment, hemodynamic stress occurs in an inside-out manner, whereas EMF occurs in an outside-in manner (Figure 3).

External cuff placement around the femoral artery in mice causes marked intimal proliferation (70). There is a case report of a young patient with idiopathic pulmonary arterial hypertension with atherosclerotic stenosis of the LAD artery due to left main trunk compression caused by aneurysmal dilation of the pulmonary trunk, despite the absence of conventional risk factors (71). Similarly, another case report showed that compression of a coronary artery by a metastatic tumor resulted in the formation of a coronary plaque within 2 months (72). These papers suggest that changes in the perivascular environment affect plaque development in an outside-in manner in both mice and humans.

Recent studies have suggested that intraplaque hemorrhage is a critical mechanism of plaque progression and rupture (7, 73), and is associated with tissue hypoxia (74), fissure, and delamination (75). We speculate that EMF is a trigger for inflammation, including hypoxia, oxidative stress, and direct tissue injury, followed by intraplaque hemorrhage.

The body, tissues, and cells are exposed by various mechanical forces, such as muscle contraction, gravity, and cardiac contraction and blood flow (76). These physical forces affect the stem cell fate (77). Vascular stem cells are derived from the adventitia (66) and also from PVAT (67, 68). EMF affecting the adventitia and PVAT, together with hemodynamic force, may induce stem cell differentiation and migration, and eventually achieve the target of remodeling. These forces may also induce phenotypic switching of macrophages (78, 79) and vascular smooth muscle cells (80). An example of this situation is that stretch-induced inflammation in macrophages has been attributed to nuclear factor-κB and NOD-like receptor family, pyrin domain containing 3 inflammasome activation (78). Therefore, at the cellular level, the PVME may induce non-resolving inflammation.

We have no precise and irrefutable evidence to prove the concept of the PVME. The limitations for conclusive evidence are as follows. First, dynamically changing properties of the PVME complicate the analysis and evaluation. Moreover, site specificity in atherosclerotic lesions between animal models and humans is not identical (6). Mouse atherosclerosis is largely observed in the extracardiac arteries, and less in the coronaries which is where humans are more susceptible (81). Therefore, experimental animal models are not appropriate for researching the pathogenesis of site specificity. Second, because the EMF in each arterial tree is different, the PVME is diverse and not uniformly determined, but is indeed site-specific. Third, the PVME varies inter-individually and intra-individually because of each anatomical property and physical activity. These differences appear to depend on several factors, such as aging, sex, physique, the atherosclerotic process, and vascular therapies. Fourth, the PVME is a determining factor of shear stress. The importance of the PVME may be obscured by shear stress itself.

## Conclusion

We propose the novel concept of the PVME. In addition to hemodynamic stress, EMF enhance atherosclerosis. The site-specific property of atherogenesis may be explained by this concept. In each vascular tree, perivascular anatomical and physical characteristics form a unique mechanical environment (i.e., the PVME). Although we discuss the pathogenesis of atherosclerosis, the concept of the PVME may be applicable to other arterial diseases, such as arterial dissection, aneurysms, and functional disorders (e.g., vasospasm and vascular remodeling). From the clinical point of view, the PVME appears to be a crucial factor of personalized medicine, such as endovascular therapies, surgical interventions, and cell-based therapies. The concept of the PVME will lead to a better understanding of vascular pathologies and the development of new therapeutic strategies for vascular diseases.

## Author contributions

TY and KM contributed to the interpretation of the available data and writing of the manuscript. Both authors contributed to the article and approved the submitted version.

## Abbreviations

ITA: internal thoracic artery
EMF: externally applied mechanical force
PVME: perivascular mechanical environment
CA: carotid artery
PVAT: perivascular adipose tissue.
